# *Rhodobacter capsulatus *porphobilinogen synthase, a high activity metal ion independent hexamer

**DOI:** 10.1186/1471-2091-5-17

**Published:** 2004-11-22

**Authors:** David W Bollivar, Cheryl Clauson, Rachel Lighthall, Siiri Forbes, Bashkim Kokona, Robert Fairman, Lenka Kundrat, Eileen K Jaffe

**Affiliations:** 1 Department of Biology, Illinois Wesleyan University, P.O. Box 2900, Bloomington, IL 61702-2900, USA; 2 Biology Department, Haverford College, Haverford, PA 19041, USA; 3 Fox Chase Cancer Center, 333 Cottman Avenue, Philadelphia, PA 19111, USA

## Abstract

**Background:**

The enzyme porphobilinogen synthase (PBGS), which is central to the biosynthesis of heme, chlorophyll and cobalamins, has long been known to use a variety of metal ions and has recently been shown able to exist in two very different quaternary forms that are related to metal ion usage. This paper reports new information on the metal ion independence and quaternary structure of PBGS from the photosynthetic bacterium *Rhodobacter capsulatus*.

**Results:**

The gene for *R. capsulatus *PBGS was amplified from genomic DNA and sequencing revealed errors in the sequence database. *R. capsulatus *PBGS was heterologously expressed in *E. coli *and purified to homogeneity. Analysis of an unusual phylogenetic variation in metal ion usage by PBGS enzymes predicts that *R. capsulatus *PBGS does not utilize metal ions such as Zn^2+^, or Mg^2+^, which have been shown to act in other PBGS at either catalytic or allosteric sites. Studies with these ions and chelators confirm the predictions. A broad pH optimum was determined to be independent of monovalent cations, approximately 8.5, and the *K*_m _value shows an acidic pK_a _of ~6. Because the metal ions of other PBGS affect the quaternary structure, gel permeation chromatography and analytical ultracentrifugation experiments were performed to examine the quaternary structure of metal ion independent *R. capsulatus *PBGS. The enzyme was found to be predominantly hexameric, in contrast with most other PBGS, which are octameric. A protein concentration dependence to the specific activity suggests that the hexameric *R. capsulatus *PBGS is very active and can dissociate to smaller, less active, species. A homology model of hexameric *R. capsulatus *PBGS is presented and discussed.

**Conclusion:**

The evidence presented in this paper supports the unusual position of the *R. capsulatus *PBGS as not requiring any metal ions for function. Unlike other wild-type PBGS, the *R. capsulatus *protein is a hexamer with an unusually high specific activity when compared to other octameric PBGS proteins.

## Background

The enzyme porphobilinogen synthase (PBGS, EC 4.2.1.24) catalyzes the first common step in the biosynthesis of the tetrapyrrole pigments such as heme, chlorophyll, and cobalamin [1]. PBGS is very highly conserved in sequence and structure but contains a remarkable phylogenetic variation in metal ion usage for catalytic and allosteric functions [2,3]. As of 2003, approximately one-half of the ~130 PBGS sequences available contained the binding determinants for a catalytic zinc ion, and about one-half did not [2]. On the other hand, approximately 90% of the known PBGS sequences contain the binding determinants for an allosteric magnesium. The only known PBGS sequences that lack the binding determinants for *both *the catalytic zinc and the allosteric magnesium are in the bacterial genus *Rhodobacter *[2]. These atypical PBGS expressed by *Rhodobacter sphaeroides *and *Rhodobacter capsulatus *were two of the earliest PBGS enzymes to be characterized in the pioneering work of Shemin and coworkers and were erroneously chosen as representative of PBGS from all photosynthetic organisms [4,5]. However, one distinct difference between the ∝-proteobacteria, of which *R. capsulatus *is an example, and other photosynthetic organisms is the biosynthetic pathway used to produce the PBGS substrate, 5-aminolevulinic acid (ALA). The ∝-proteobacteria synthesize ALA from succinyl-CoA and glycine while other photosynthetic organisms use glutamic acid to make ALA [6].

In light of the vast information now available on phylogenetic variations in tetrapyrrole biosynthesis and on the PBGS that require a catalytic zinc and/or that utilize an allosteric magnesium, the current study revisits the PBGS of *Rhodobacter capsulatus *with emphasis on understanding the enzyme's unique characteristics. Since other PBGS have been shown to absolutely require divalent cations for catalytic activity, and in light of the enhanced purity of modern reagents, it is important to revisit the metal ion requirements of *R. capsulatus *PBGS to test the predictions of the sequence analysis that suggests the absence of any metal binding determinants. Herein we present evidence that there is absolutely no effect of Zn^2+ ^or Mg^2+ ^on the activity of the enzyme and no other metal ions appear to be required for enzyme function. Prior studies have also shown that some PBGS enzymes exhibit a pH rate profile whose pKa value is altered by the presence of monovalent cations [7,8]. Hence, we include an analysis of enzyme activity in relation to pH and monovalent cations.

The native holoenzyme quaternary structure for PBGS from most species is a homo-octamer as supported by 18 deposited PBGS crystal structures from yeast, human, *E. coli*, and *Pseudomonas aeruginosa*, and noncrystallographic cross-linking data on PBGS from the green plant pea [9-14]. However, an alternative hexameric structural variant was revealed by the crystal structure of a rare allele of human PBGS [15]. The hexameric structure suggests a functional relationship between binding of the allosteric magnesium of most PBGS and a putative hexamer-octamer distribution that serves as the structural basis for allosteric regulation of enzyme function [15]. In the absence of the binding sites for either catalytic or allosteric metal ions we investigated the oligomeric structure of *R. capsulatus *PBGS, and results suggest that the protein is a homo-hexamer. We present a homology model of the hexameric *R. capsulatus *PBGS structure.

## Results

### Cloning and sequencing

**Figure 1:**
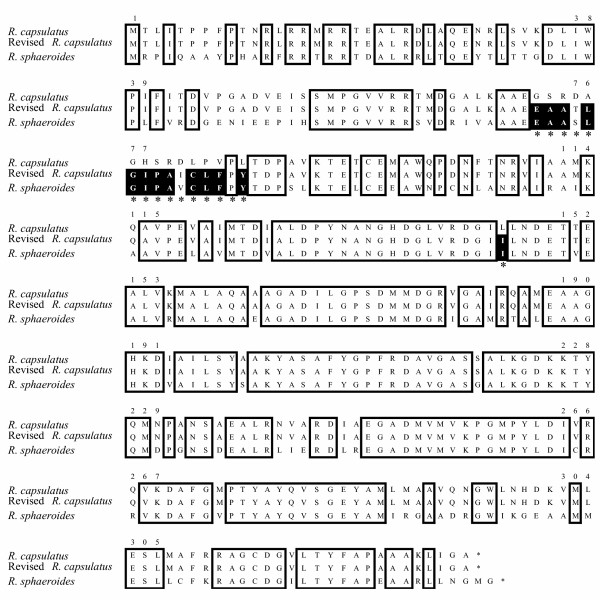
**PBGS Protein Sequence Alignment. **Amino acid sequences as predicted from published DNA sequences for *R. capsulatus *PBGS and *R. sphaeroides *PBGS as well as the PCR product sequences from our genomic *R. capsulatus *DNA (labeled as revised sequence). Sequence identity between all three sequences is boxed. The regions where the published *R. capsulatus *sequence and the sequence we determined differ are indicated with an asterisk below those amino acid positions. In these regions, a black background indicates where sequence matches occur between the revised sequence for *R. capsulatus *PBGS and *R. sphaeroides *PBGS, but not for the previously published *R. capsulatus *PBGS sequence.

The cloning of the *hemB *gene (which encodes PBGS) from *R. capsulatus *was accomplished by PCR using primers based upon the sequence of Indest and Biel [[16], GenBank accession U14593]. The sequence of the cloned gene differed from that published previously. Figure 1 presents an alignment of the predicted polypeptides based on the published *R. capsulatus *and *R. sphaeroides *sequences (GenBank accession number AAL 26883) as well as the sequence determined from our PCR product (GenBank accession number AY618996). The changes in the predicted polypeptides are due to three differences that we observe when comparing the nucleotide sequence determined herein with the published sequence: a deletion of G at position 425, an insertion of an A at position 468, and reversal of the AC at positions 643–644 to CA. The first two sequence differences result in alteration of the predicted amino acid sequence from amino acids 72 to 86. The third sequence difference alters the amino acid at position 145 from leucine to isoleucine. Based on the aligned polypeptide sequence, the newly determined sequence appears more homologous to *R. sphaeroides *PBGS, and hence is deduced to be the correct sequence.

### Expression and purification

**Figure 2:**
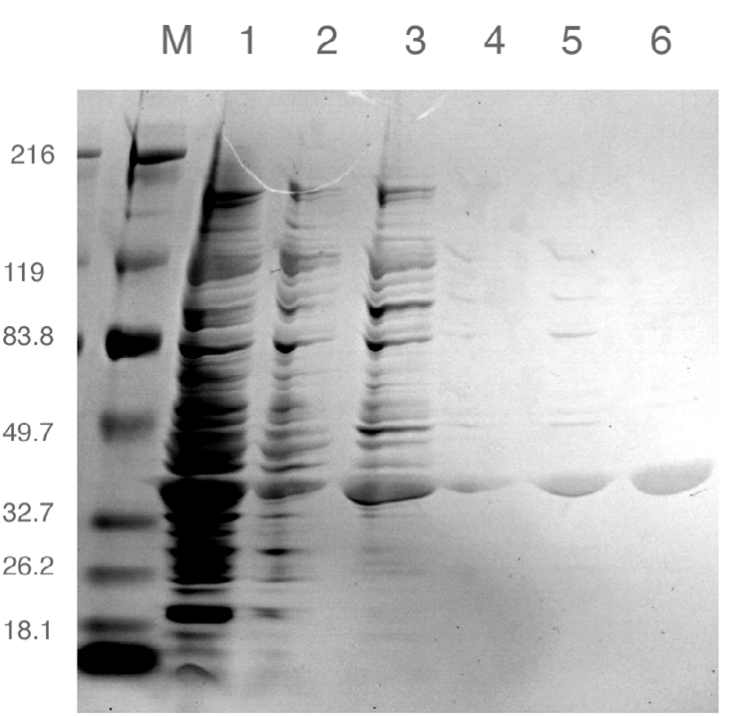
**SDS-PAGE (4–20%) of PBGS Purification Steps. **Lane M is marker; lane 1 is Crude Extract; lane 2 is Ultracentrifuge supernatant; lane 3 is 25% ammonium sulfate pellet; lane 4 is pooled Phenyl-Sepharose fractions; lane 5 is pooled DEAE fractions; and lane 6 is pooled S-300 fractions.

As demonstrated in Figure 2, we were able to express and achieve substantial purification of *R. capsulatus *PBGS. The specific activities of the ultracentrifuge supernatant, redissolved ammonium sulfate pellet, phenyl-sepharose pool, DEAE pool, and concentrated S-300 pool were 130, 250, 212, 176, and 364, μmol h^-1 ^mg ^-1 ^respectively. We suspect that some of this variation is due to a protein concentration dependence to the specific activity (see below). The enzyme has an apparent molecular weight that is in agreement with the predicted mass of 35.8 kDa (Figure 2). Based on SDS-PAGE and silver staining, the protein appears to have been purified to homogeneity.

### Protein concentration dependence of the specific activity

**Figure 3:**
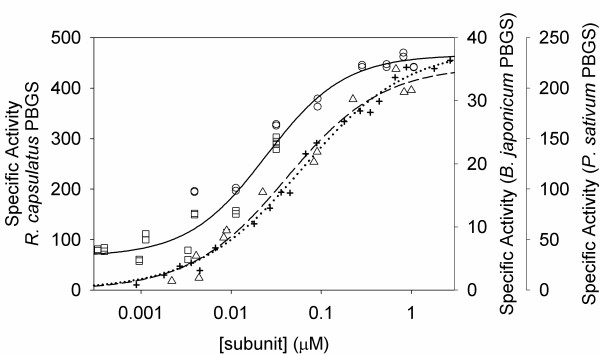
**Protein Concentration Dependence to *R. capsulatus *PBGS Specific Activity. **Protein was varied from 0.01 μg ml^-1 ^to 40 μg ml^-1 ^and assay times used were 5 min (○) and 1 h (□) with the purified *R. capsulatus *enzyme. The solid line represents a hyperbolic fit to the activity data presented for the combined 5 min and 1 h assays. The final pH for the *R. capsulatus *assays was 8.3 in BTP-HCI. For comparison, protein concentration curves are presented from previous experiments for *B. japonicum *PBGS (+) [7] and *P. sativum *PBGS (Δ,) [9] and the hyperbolic fits are presented as dotted and dashed lines respectively.

Other PBGS that lack the catalytic zinc ion binding site have been shown to exhibit a protein concentration dependence to the specific activity [7-9] This unusual phenomenon indicates that a maximally active oligomer can dissociate into less active smaller units. A protein concentration dependent specific activity is illustrated for *R. capsulatus *PBGS in Figure 3. The maximal *R. capsulatus *PBGS activity, ~450 μmol h^-1 ^mg ^-1^, is the highest ever seen for a purified PBGS, and the lowest *R. capsulatus *PBGS activity, ~150 μmol h^-1 ^mg^-1^, shows that the smallest oligomeric structure retains significant activity. As shown in Figure 3, the activity of the smaller unit is significantly different from what is documented for PBGS that lack the catalytic zinc but contain the allosteric magnesium binding site; in those instances, the smallest oligomers are inactive [7,9]. Kinetic characteristics described below use protein concentrations varying from 0.15–15 μg ml^-1 ^and this variation does not appear to effect the pK_a _values apparent from the pH rate profiles, the *K*_m _values, or the effects of metal ions or metal ion chelators, which suggest that these properties are more or less the same for the largest and smallest oligomer.

### Kinetic characteristics

Metal ion requirements of purified *R. capsulatus *PBGS were determined and the results confirm that the *Rhodobacter *enzyme is different in its response to a variety of cations compared to most known PBGS enzymes. At a protein concentration of ~1 μg ml^-1 ^there is no significant stimulation or inhibition of *R. capsulatus *PBGS by the addition of Zn^2+ ^or Mg^2+ ^ions. The presence of Zn^2+^, up to 100 μM, caused no change in activity and inclusion of 10 mM Mg^2+ ^resulted in 86% activity. There is also no apparent inhibition by the addition of 10 mM EDTA or by pretreatment with Chelex resin. Inclusion of 1,10-phenanthroline from concentrations of 10 μM – 10 mM had no effect on *R. capsulatus *PBGS activity. The purified enzyme was tested for the presence of zinc and magnesium by atomic absorption spectroscopy; under conditions where it would have been possible to detect as little as 0.05 metal ion per subunit, none were detected.

**Figure 4:**
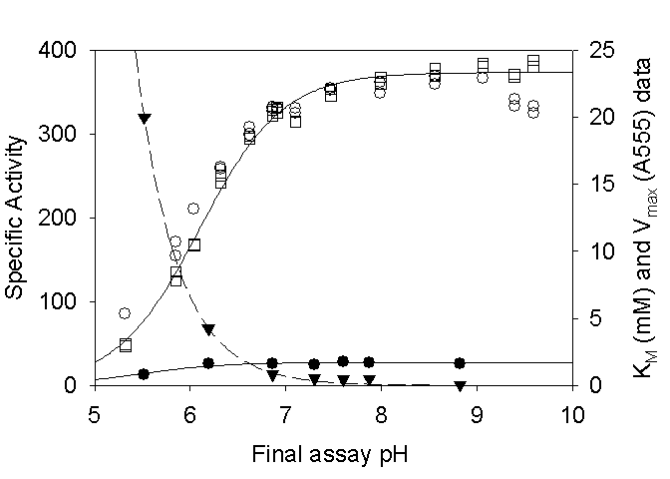
**pH Rate Profile. **The pH rate profile is presented at 10 mM ALA in BTP-HCl in the presence (○) and absence (□) of 0.1 M KCl. Protein was 15 μg ml^-1^. The *K*_m _(filled tringle) and V_max _(filled circle) values as a function of pH at (0.15 μg ml^-1^) are given in units of mM and A_555_, respectively.

Previous experiments with a wide variety of PBGS enzymes suggest that monovalent cations can affect the activity of the enzyme. Such cations were clearly shown to shift the pH rate profile for some PBGS [7,8]. Results presented in Table I (data obtained at ~1 μg protein ml^-1^) suggest that there is little if any effect of the monovalent cations K^+^, Na^+ ^or NH_4_^+ ^over a wide range of concentrations (0–100 mM). Figure 4 shows that inclusion of 0.1 M KCl has no significant effect on the pH rate profile at 15 μg ml^-1 ^protein concentration.

**Table 1:** Monovalent cation effects. Samples were pre-incubated with various concentrations of chloride salts of the monovalent cations and then assayed using the standard procedure. Reported values and standard errors are triplicate absorbances at 555 nm.

	**Concentration of Salt **(mM)					
	0	1	5	10	50	100

KCl	0.270 ± 0.019	0.268 ± 0.006	0.271 ± 0.002	0.261 ± 0.007	0.269 ± 0.003	0.310 ± 0.014
NaCl	0.302 ± 0.006	0.294 ± 0.017	0.292 ± 0.002	0.276 ± 0.012	0.262 ± 0.003	0.268 ± 0.003
NH_4_Cl	0.297 ± 0.022	0.278 ± 0.004	0.279 ± 0.009	0.225 ± 0.001	0.224 ± 0.009	0.220 ± 0.005

To determine the optimal pH for the enzyme we determined the *V*_max _and *K*_m _values at ~1 μg ml^-1 ^protein at a variety of pH values as presented in Figure 4. The results demonstrate that maximal activity was observed around pH 8.0, but the enzyme is still very active over a wide range of pH values. It is also clear that the *K*_m _value drastically increases at lower pH values, which is similar to what has been observed for other PBGS enzymes *e.g.*, human PBGS [17], *E. coli *PBGS [18], or *B. japonicum *PBGS [7]. Based on the references cited, the rise in *K*_m _at low pH appears to be independent of the metal ion requirements for PBGS.

### Quaternary structure

**Figure 5:**
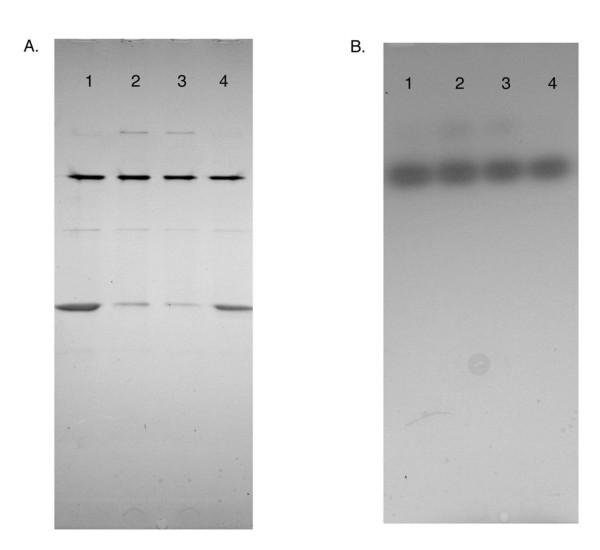
**Native gel electrophoresis. **Part A presents the Coomassie stained gel, part B presents the same gel stained with an activity stain based on the pink color formed by complex of porphobilinogen with Ehrlich's reagent. Lanes 2 and 3 contain enzyme preincubated with ALA, Lanes 1 and 4 do not include ALA in the preincubation buffer. Each lane was loaded with 2.25 μg of protein.

A recent paper described an alternative quaternary structure for a rare allele of human PBGS. The unprecedented structural change was shown to have significant effects on the enzyme activity [15]. In contrast to the active octameric human PBGS, a hexameric form observed with the rare human allele was relatively inactive. The interconversion of these two oligomeric structures was related to the allosteric regulation of some non-human PBGS by magnesium. To assess the different oligomerization states possible for *R. capsulatus *PBGS, a native gel analysis was performed and the gel was stained for enzyme activity followed by Coomassie staining for protein (see Figure 5). Previous studies have shown that native gel electrophoresis can give good separation of the quaternary structure forms of PBGS (see also ref [19]). Four different sized complexes are observed whose mobility fit well to an mixture of dimer, tetramer, hexamer, and octamer (note constant charge/mass ratio). As had been seen before for *E. coli *PBGS [19], the distribution of these oligomeric forms is altered by substrate. By comparing samples preincubated without substrate (Figure 5A, lanes 1 and 4) with samples preincubated in the presence of substrate (Figure 5A, lanes 2 and 3) it can be observed that the complex that runs as the smallest form (putative dimer) becomes less prominent and the largest molecular weight complex (putative octamer) becomes visible. It is also interesting to note that no activity is observed for the two smallest complexes (Figure 5B).

Based upon a combination of size-exclusion chromatography and analytical ultracentrifugation, the size of the major complex was determined. The major component in size exclusion chromatography ran at an approximate molecular weight of 220,000 Daltons. The predicted molecular weight for a monomer is 35,856 Daltons so it would appear that *R. capsulatus *PBGS is predominantly a hexamer.

Ultracentrifugation data for samples collected at several speeds were fit well using a single species model and show that the *R. capsulatus *PBGS is largely a hexamer (Table II). The molecular weight obtained from a global fit to data collected at 8,000, 10,000, 12,000 and 14,000 rpm is 215,700 ± 9,700 and was largely independent of speed, indicating strong evidence for ideal single species behaviour. Since we do observe a slight trend in decreasing molecular weight with increasing speed, we further analyzed the data by considering more complex two state models (Table III). The lowest apparent molecular weight from Table II was no smaller than that expected for a hexamer so we mainly considered models with the hexamer and some larger second state. A lack of lower molecular weight species is not surprising since the protein concentration dependence of the activity plateaus at about 1 μM and the loading concentration in the AU is greater than ten-fold higher. As judged by the square roots of variance for the fits of the various models to the data, collected either in dithiothreitol (DTT) or 2-mercaptoethanol (βME), we saw no appreciable improvement in the fits relative to the pure hexamer model. In the one instance where a slight improvement was noted (the hexamer-octamer model for the βME sample), less than 5% of the total absorbance could be ascribed to the octamer, suggesting very little higher order self-association. Prolonged dialysis results in some non-specific aggregation, presumably due to a build-up of oxidized DTT or βME, and may provide an explanation for problems with unwanted aggregation. Finally, we also tested the possibility that the apparent molecular weight from single species analysis might instead reflect a proportionation between an octamer and some smaller species. We were able to rule this out as such models resulted in poorer fits to the data (e.g. a fit to a tetramer-octamer model is shown in Table III).

**Table 2:** Molecular weight analysis of *R. capsulatus *PBGS as measured by equilibrium sedimentation. Data were collected at 4°C. The column headings refer to RPM values. All results are in Da. The monomer molecular weight is 35,857 Da.

Sample	**8000**	**10000**	**12000**	**14000**	**Overall**
**12.3 μM (.1 mM DTT)**	221,698	222,291	213,584	206,743	215,700 ± 9,700
**12.3 μM (1 mM βME)**	227,785	230,906	224,189	205,239	220,200 ± 9,100

**Table 3:** Sedimentation equilibrium model analysis of *R. capsulatus *PBGS. All numbers reported are the square root of variance (×10^-3^) from the fits of the various models to the data. The data for all speeds were fit globally to individual models

**Model**	**(.1 mM DTT)**	**(1 mM BME)**
**single species**	10.50	8.20
**hexamer**	10.49	8.38
**6----->8**	10.51	8.27
**6----->10**	10.52	8.32
**6----->12**	10.54	8.36
**4----->8**	12.98	9.82

### *R. capsulatus *PBGS hexamer homology model

**Figure 6:**
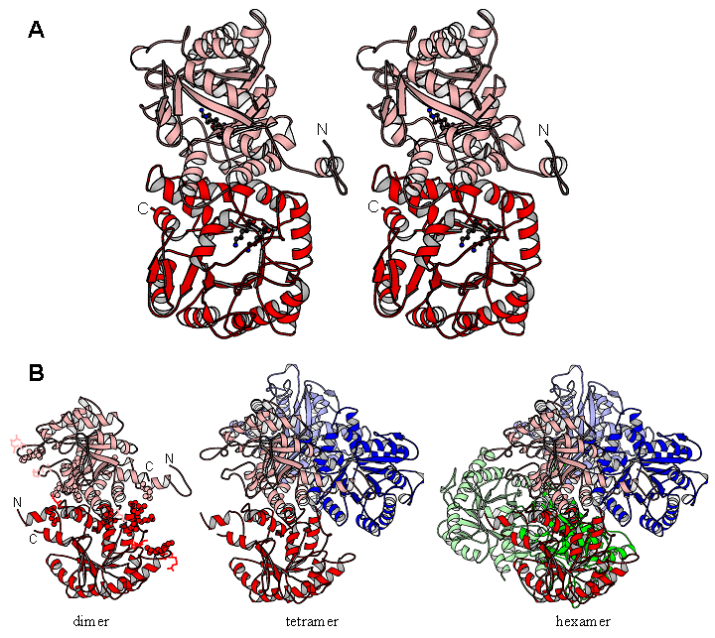
**A homology model for hexameric *R. capsulatus *PBGS. **A) The *R. capsulatus *PBGS detached dimer model, shown in stereo view, looking down the αβ-barrel domain of subunit A (red), directly into the active site. The two active site lysine residues, implicated in covalent catalysis [13], are shown ball-and-stick. The N and C termini are labeled for subunits B and A respectively. B) The hexamer is composed of three detached dimers, which are shown as ribbon representations and colored shades of red, blue, and green. For the dimer view only, side chains in regions where the model must accommodate deletions and/or insertions are shown as sticks colored as the subunit; amino acids homologous to, but different from those at the hugging dimer interface of *P. aeruginosa *PBGS are shown in space fill representation. In this representation, the N and C termini are visible for subunits A and B and are labeled in the dimer representation.

Figure 6 is an illustration of the homology model of hexameric *R. capsulatus *PBGS based on a model of hexameric *P. aeruginosa *PBGS (see below). Regions of highest uncertainty are places where alignment of *R. capsulatus *PBGS and *P. aeruginosa *PBGS contain insertions and deletions. These regions are illustrated in the Figure; they are all on the solvent exposed surface of the oligomer and thus unlikely to affect subunit interactions. In an attempt to understand why, unlike other PBGS, *R. capsulatus *PBGS does not predominate as an octamer, we analyzed points of subunit contact in the octamer of the highly homologous *P. aeruginosa *PBGS. Each *P. aeruginosa *PBGS monomer contains thirty-six residues that are within 3.2 Å of an adjacent subunit of the octamer. Twenty-five of these residues are identical in *R. capsulatus *PBGS; eleven are different. The majority of these differences result in a reduced ability in *R capsulatus *PBGS to form an ion pair or hydrogen bonding interaction at the subunit interface. The differences are tyrosine to phenylalanine or isoleucine, arginine to glutamine or glycine, valine to isoleucine, aspartic acid to asparagine or alanine, histidine to arginine or leucine, and glutamic acid to alanine or asparagine. This analysis is consistent with the observation that the interactions between PBGS subunits are largely hydrophilic in character with ordered water molecules at the subunit interfaces (see Discussion).

## Discussion

Prior to the current work, it appeared that there was a considerable sequence diversion in a 13 amino acid segment between *R. capsulatus *and *R. sphaeroides *PBGS. The revised sequence for *R. capsulatus *PBGS presented shows close homology between the PBGS of the two *Rhodobacter *species. This is interesting in light of the apparent differences between these enzymes regarding monovalent cation usage. The *R. sphaeroides *enzyme is reported to be stimulated by monovalent cations [5], while the *R. capsulatus *is not affected by monovalent cations. While it was previously tempting to ascribe the difference in response between the enzymes to this region, clearly this is not the case.

To further analyze the enzymatic characteristics of the *R. capsulatus *PBGS, the enzyme was purified after being heterologously expressed in *E. coli*. The purification has allowed us to carry out definitive experiments on the requirements for *R. capsulatus *PBGS function.

Based on sequence comparisons and known crystal structures for some PBGS, the *Rhodobacter *PBGS appear to constitute a unique form of the enzyme that does not require metal ions for structure, activity, or allosteric regulation [2]. Although the original description of the *Rhodobacter *PBGS enzymes [4,5] did not demonstrate a requirement for metal ions, the reagents of that time period are now known to be contaminated with metal ions, particularly zinc. The current reagents are of better quality, thus allowing us to confirm the metal requirements for *R. capsulatus *PBGS. *R. capsulatus *PBGS activity is independent of all metal ions tested.

It has been proposed that chloroplast containing photosynthetic organisms use the allosteric regulation of PBGS by magnesium as part of a complex control of the biosynthesis of chlorophyll [15]. Although *Rhodobacter capsulatus *makes a similar pigment, bacteriochlorophyll, the PBGS enzyme from this organism does not exhibit any regulation by divalent cations. In the absence of the allosteric magnesium, *Rhodobacter *must use alternative mechanisms to prevent the accumulation of photoreactive intermediates in the biosynthesis of its physiologically relevant tetrapyrroles.

The preferred pH for enzyme function was determined by measuring *V*_max _and *K*_m _in a systematic fashion. Based upon these analyses, the apparent pH optimum is approximately pH 8.0, but the enzyme demonstrates significant activity from pH 7–9. The *K*_m _value at optimal pH is still high (0.5 mM) relative to other PBGS at their optimal pH in the presence of their required metal ions (~150 μM). This suggests that an unknown factor may be required *in vivo*. Because there have been reports for stimulation of PBGS enzymes from other organisms by the addition of monovalent cations, several monovalent cations were tested for their ability to stimulate enzyme function at the pH optimum. At the pH optimum no stimulation by monovalent cations was observed (see Figure 4).

The rare human allele for PBGS encoding F12L revealed the possibility for alternative quaternary structures of PBGS that have been proposed to form the basis for allosteric regulation of PBGS that contain an allosteric magnesium binding site [15]. These types of PBGS are found in all plants, all archaea, and all bacteria except those of the genus *Rhodobacter *[2]. Evidence for alternate quaternary forms of PBGS is particularly apparent for those PBGS that contain the allosteric magnesium but do not contain the catalytic zinc, because these forms exhibit a protein concentration dependence to their specific activity as illustrated in Figure 3[7,9,15]. The protein concentration dependence has been proposed to be due to an equilibrium between a fully active octamer and an inactive hexamer [15], which is consistent with the data for pea and *B japonicum *PBGS presented in Figure 3. The data for *R. capsulatus *PBGS suggests however that the active form of this protein is a hexamer of specific activity ~400 μmol h^-1 ^mg^-1^, and that this active hexamer can dissociate into a smaller form that is less active, but not inactive, with a specific activity of ~150 μmol h^-1 ^mg^-1^. These observations lead to three interrelated questions. Why does *R. capsulatus *PBGS associate into a hexamer rather than an octamer; why is the *R. capsulatus *PBGS hexamer of high activity rather than of low activity; and what is the less active quaternary structure that is in equilibrium with the *R. capsulatus *PBGS hexamer?

To answer these questions, we need to address the factors that govern the interconversion of PBGS quaternary forms and the structural basis for the different activities associated with these different quaternary forms. Crystal structures reveal that those PBGS that readily interconvert between quaternary forms contain subunit-subunit interfaces that are hydrophilic in character. For instance, in the *P. aeruginosa *PBGS structure, the interaction of the barrel of subunit A and the N-terminal arm of subunit B, which form a hugging interaction, is dominated by hydrogen bonds and buried water molecules. One could argue that this type of subunit-subunit interface can readily dissociate because the protein-protein interactions are similar in energy to aqueous solvation of the protein surfaces [20,21]. A sequence comparison between *P. aeruginosa *PBGS and *R. capsulatus *PBGS shows that the amino acids that lie at the hugging dimer interface of the former are of lower hydrogen bonding and ion pairing capacity in the latter, which might explain why *R. capsulatus *PBGS is not an octamer. As for the functional difference between human octameric and hexameric PBGS, this has been ascribed to the mobility/stability of an active site lid that serves to gate access to solvent [15]. In that case, destabilization of the lid causes the pH rate optimum for the reaction to shift dramatically toward basic pH and causes a dramatic decrease in affinity for the *K*_m _determining substrate molecule since the lid residues form part of the binding site for this substrate [15]. Consistent with the fact that *R. capsulatus *PBGS appears to be a hexamer, the pH optimum is basic and the *K*_m _is 2 – 3 fold higher than most other PBGS species under their optimal assay conditions. Crystallization trials for *R. capsulatus *PBGS are currently underway to provide insight into these fascinating questions. Finally, based on the native gel analysis, we propose that the active *R. capsulatus *PBGS hexamer can dissociate into a less active dimer. Preliminary unpublished results suggest that one can produce an active dimeric species of human PBGS by destabilizing the dimer-dimer interactions that are essential for oligomerization of PBGS into either the hexamer or the octamer.

## Conclusions

The evidence presented in this paper supports the unusual position of the *R. capsulatus *PBGS as not requiring any metal ions for catalytic function, which may be characteristic of the *Rhodobacter *genus. Unlike other wild-type PBGS, the *R. capsulatus *protein is a hexamer. What remains to be determined is how the reaction mechanism for this enzyme might differ from those PBGS that show both an absolute requirement for metal ions and an octameric quaternary structure.

## Methods

### Materials

Chemicals and buffers were obtained from Fisher or Sigma, in the purest possible form, except where noted below. Ultrafiltration devices used for concentrating protein were obtained from Fisher as were Slide-A-Lyzer dialysis cassettes.

### Construction of the expression plasmid

The DNA encoding PBGS was amplified from *R. capsulatus *genomic DNA by PCR using oligonucleotide primers (Integrated DNA Technologies) directed to the 5' and 3' ends of the coding region based on published sequence [16]. The forward primer PBGS 5' (5'-GCATATGACCCTGATCACCCCCCCC-3') introduced an *Nde*I site and the reverse primer PBGS 3' (5'-CGGATCCGCGGTCAGGCGCCGATCAGC-3') introduced a *Bam*HI site. The PCR reaction was performed using a thermocycler from MWG Scientific and Pfu polymerase (Stratagene) with the following program: 45 sec at 95°C, 45 sec at 48°C, 1 minute at 72°C. The resulting PCR fragment was cloned into vector pPCRScript Amp (Stratagene) creating plasmid pPBGS1. The PCR fragment was removed from the vector by digestion with *Nde*I and *Bam*HI and ligated into the vector pET11a (Novagen) digested with the same restriction enzymes. The resultant plasmid pPBGS4 was sequenced in the FCCC DNA Sequencing Facility using external and internal primers to confirm the sequence in both directions. For expression, the recombinant plasmid pPBGS4 was transformed into strain BLR (DE3).

### Enzyme expression and purification

A 1 L culture of LB broth with 0.4% glucose was inoculated with a single colony from a fresh transformation and grown for 16 hours at 37°C. The cells were harvested by centrifugation (10 min at 10,800 × *g*) and resuspended in fresh LB medium containing no glucose, but with 100 μM isopropyl-1-thio-β-D-galactopyranoside (Research Organics) and grown for 45 hr at 15°C. From this point all steps were performed on ice or at 4°C. Cells were harvested by centrifugation for 10 min at 10,800 × *g *with a yield of 5.74 g wet weight. The cells were washed with 0.1 M BisTris Propane (BTP, Research Organics) pH 8.5 and then resuspended in 15 ml of 0.1 M BTP pH 8.5 and lysed by two passes through a French Press in the presence of Benzonase™ (Novagen) nuclease and 1 mM AEBSF (Calbiochem). Unbroken cells, inclusion bodies, and debris were removed by centrifugation for 15 min at 21,500 × *g*. The sample was then ultracentrifuged for 1 hour at 141,000 × *g *to remove membranes. The enzyme was precipitated from solution by treatment with 25% ammonium sulfate and collected by centrifugation for 20 min at 31,000 × *g*. The pellet was resuspended in 0.1 M BTP pH 7.0 and loaded onto a 100 ml Phenyl-Sepharose (Amersham Biosciences) column pre-equilibrated in 20% ammonium sulfate, 0.1 M BTP pH 7.0 buffer. The enzyme was eluted from the column with a two column volume gradient running from 20% to 0% ammonium sulfate in 30 mM BTP pH 7.0. The PBGS eluted from the column very close to the end of the gradient. Fractions with specific activity greater than 100 μmol h^-1 ^mg^-1 ^were then pooled and loaded to a 100 mL DEAE-Sepharose column equilibrated in 30 mM BTP pH 7.5 and eluted with a two column volume gradient from 0 to 0.4 M KCl in 30 mM BTP pH 7.0. The fractions with a specific activity greater than 300 μmol h^-1 ^mg^-1 ^eluted near the end of the gradient and were pooled and concentrated using centrifugal concentrators. The concentrated DEAE fraction (0.9 mg ml^-1^) was then loaded on to a 300 mL S-300 column (2.6 cm × 60 cm) (Amersham Biosciences) pre-equilibrated with 0.1 M BTP pH 7.0, and eluted with the same buffer. The fractions with specific activity greater than 185 μmol h^-1 ^mg^-1 ^were pooled and concentrated. The enzyme appeared to be greater than 95% pure based on SDS-PAGE analysis.

### Enzyme activity assays

Enzyme was pre-incubated in 0.1 M BTP pH 8.6 in the presence or absence of various metal ions and other reagents for 10 min at 37°C. Assays were initiated by the addition of ALA-HCl (Aldrich) to a final concentration of 10 mM and were allowed to run for 5 minutes in a final volume of 1 ml. Assays were terminated by the addition of 0.5 ml of 20% TCA. The product was then quantified by reaction of the stopped assay mixture with an equal volume of modified Ehrlich's reagent and measurement of absorbance at 555 nm approximately 8–10 minutes later. All assays were performed in duplicate or triplicate. If the amount of product resulted in an absorbance above 1.0 OD, the stopped assay mixture was diluted prior to the addition of Ehrlich's reagent. Inhibition by 1,10-phenanthroline was carried out as described previously [22]. Inhibition by Chelex 100 resin (BioRad) was assayed by incubating PBGS in 0.1 M BTP pH 8.5 at 0.05 g resin per mg enzyme, on ice for 4 hours with occasional stirring, followed by centrifugation at 13,000 × g for 5 min to pellet the resin. The resultant enzyme was then assayed. For determination of monovalent cation effects at pH 8.3, the enzyme was first dialyzed against 100 mM BTP pH 8.6. Monovalent cations were added as the chloride salts.

### Effect of pH

For assays performed as a function of pH, the buffer was 0.1 M BTP (initial pH 6–9). For determination of *K*_m _and *V*_*max *_as a function of pH, the enzyme was at 0.13 μg ml^-1^. Since the substrate is an acid, the actual pH values for assays were determined by running a mock assay without enzyme and measuring the actual pH of the combined reaction. When ALA concentration was varied for determination of *V*_max_, the pH of the assay mixture was adjusted with 0.5 M HCl to control the final pH. For determination of the pH rate profile with and without 0.1 M KC1, the enzyme was at 15 μg ml^-1^.

### Gel electrophoresis

SDS-PAGE was performed using the Laemmli system and precast 4–20% gradient gels from Cambrex (Rockland, ME). Gels were stained using a silver stain kit from Pierce Chemical (Rockford, IL). Native gels were performed using the same gel system but omitting SDS from all buffers. Activity staining of the gel was performed as described previously [19]. Following a wash with 20% TCA, the activity stained gel was then stained with Coomassie blue to visualize protein bands. Samples for the native gels were preincubated at a final concentration of 0.15 mg ml^-1 ^for 10 minutes in 0.3 M Tris pH 6.8 with additions of 100 μM Zn, 13 mM ALA or both prior to loading. 15 μl of the preincubated sample was then loaded to each well.

### Holoenzyme size determination

The size of the enzyme was determined both by size exclusion chromatography and by analytical ultracentrifugation (AU). Size exclusion chromatography was performed using a Waters 600 system equipped with a Waters 996 photodiode array for detection of protein elution at 280 nm. The column used was a Superose 6 (10 × 300 mm, Amersham Pharmacia) and was run at 0.5 ml/min with 100 mM BTP pH 8.6 with 100 μL of a 200 μg ml^-1 ^solution. The column was calibrated with standards obtained from the manufacturer. For AU experiments, protein samples were dialyzed against 10 mM Tris-HCl pH 7.7 with either 0.1 mM DTT or 1 mM βME as reducing agent. Although TCEP (Tri(2-carboxyethyl)phosphine) is the preferred reducing agent for such biophysical experiments, we discovered that the oligomeric state was destabilized in the presence of this reagent with other PBGS; weak chelation of divalent cations has been observed for this reagent suggesting a mechanism for this destabilization. Protein loading concentrations were 12.3 μM in monomer (440 μg ml^-1^). Concentrations were determined by UV absorption at 280 nm. The extinction coefficient used for the protein was 29,870 L mol^-1 ^cm^-1 ^and represents the sum of individual tyrosine and tryptophan absorbance coefficients. Sedimentation equilibrium (SE) experiments were carried out at 4°C using a Beckman Optima XL-A analytical ultracentrifuge equipped with an An60 Ti four-hole rotor. Samples were loaded into six-channel charcoal-filled Epon centerpieces. Temperature-corrected partial specific volumes and solution densities were calculated using the Sednterp program [23]; the solution density was 1.00028 g ml^-1 ^and partial specific volume was 0.7304 ml g^-1^. Data analysis was performed using the WinNonlin V.1.060 nonlinear least squares fitting program obtained from the National Analytical Ultracentrifugation Facility at the University of Connecticut.

### Homology Model Building

The only existing crystal structure on which one can base a model of hexameric *R capsulatus *PBGS is that of hexameric human PBGS clinical variant F12L, PDB code 1PV8 [15]. Unfortunately, the crystal structure of F12L shows significant disorder, which limits its use as the sole foundation for homology model building. However, comparison of human PBGS octameric and hexameric structures (PDB codes 1E51 and 1PV8) show near identity for the amino acids that comprise a TIM-like αβ-barrel domain. The differences between the octamer and the hexamer are in the 24 N-terminal amino acids and in the disordered regions [15]. Hence, one can use a higher quality crystal structure of a PBGS octamer for homology model building the αβ-barrel domain of *R. capsulatus *PBGS. The chosen structure is PDB code 1GZG [13], which is a highly ordered, high resolution crystal structure of *Pseudomonas aeruginosa *PBGS, and 56% sequence identical to *R. capsulatus *PBGS. The model building procedure was a two step process. The first step was construction of a model of a hexameric form of *P. aeruginosa *PBGS; the second step was to use that hexamer to build the *R. capsulatus *PBGS hexamer.

*P. aeruginosa *PBGS hexamer preparation used various capacities of the program Swiss-PDB Viewer [24] and some in-house programs. First, the N-terminal arms (amino acid numbers <32) were removed from the structure file for the 1GZG dimer. The resulting αβ-barrel domains were successively overlaid upon the three dimers of hexameric 1PV8 to create a hexameric assembly of *P. aeruginosa *PBGS αβ-barrels. There is no significant sequence identity between the N-terminal arms of human and *P. aeruginosa *PBGS, hence there is an alignment ambiguity when trying to build the outstretched arms of the *P. aeruginosa *PBGS hexamer. However, there is a region of the arm that is α-helical in both the human octamer and the human hexamer. Hence, a structure alignment of octameric forms of human PBGS and *P. aeruginosa *PBGS was used to determine the proper sequence alignment for this α-helical segment. This information was used to spatially position the amino acids 22 – 29 of *P. aeruginosa *PBGS in the hexamer according to the position of this helix in the hexamer of human PBGS. Loop and side-chain prediction was performed in a graphical user environment, developed in the FCCC Molecular Modeling Facility, that integrates the functions of the programs Loopy [25], and SCWRL [26]. Within this environment, the program Loopy [25] was used to model the backbone of amino acids 29 – 32, so as to connect the N-terminal α-helix to the αβ-barrel domain of each subunit. The most N-terminal amino acids were built onto the structure within the Swiss-PDB viewer software using phi, psi, and omega angle information for the corresponding amino acids of hexameric human PBGS. Finally, the program SCWRL [26] was used to position the side chains for the N-terminal arm segments resulting in a model for hexameric *P. aeruginosa *PBGS, which could then be used for preparing hexameric models of other PBGS as we have done before for the octameric forms of pea and *D. melanogaster *PBGS [7,9]. The model for hexameric *R. capsulatus *PBGS was built using the same integrated graphical environment developed in house. This software integrates sequence alignment, threading, loop model building to accommodate insertions and deletions, and side chain optimization similar to that used for our previously published models [7,9].

## Authors' contributions

DWB was responsible for the initial cloning by PCR, purification of the enzyme, initial assays of the enzyme, and preparation of Figs. 1, 2, and 5. CC created the expression vector and helped with initial purification and assays. RL was involved in determining the pH dependence of the enzyme. SF performed the native gel electrophoresis experiments and biochemical characterizations. BK and RF performed the analytical ultracentrifugation experiments. LK and EKJ were involved in creating the homology model. EKJ performed much of the writing, supervised some of the biochemical characterization and prepared Figs. 3, 4, and 6.
